# Closure of round cutaneous defects progressively with the purse string suture technique

**DOI:** 10.11604/pamj.2015.22.16.7378

**Published:** 2015-09-09

**Authors:** Fatih Küçükdurmaz, Ismail Agir, Seyitali Gümüstas, Hakan Kivilcim, Cihangir Tetik

**Affiliations:** 1 Department of Orthopaedics and Traumatology, Faculty of Medicine, Bezmialem Vakif University, 34093 Istanbul, Turkey; 2 Department of Orthopaedics and Traumatology, Faculty of Medicine, Adiyaman University, 02040 Adiyaman, Turkey; 3 Department of Orthopedics and Traumatology, Umraniye Research and Education Hospital, 34899 Istanbul, Turkey; 4 Department of Orthopedics and Traumatology, Umraniye Research and Education Hospital, 34899 Istanbul, Turkey

**Keywords:** Purse string suture technique, skin defect, tissue expander, primary healing, secondary healing, round defects

## Abstract

**Introduction:**

There are many closure techniques available to cutaneous surgeons. One of them is the purse-string suture which is used to provide complete or partial closure of round skin defects. In our animal study; we closed skin defects with using subcuticular purse string suture technique by progressively cinching wound and we aim to more rapidly healing according to secondary healing.

**Methods:**

After anaesthetize, we created a 4 cm diameter circular full thickness skin defect on dorsal area of rats. In group 1, subcuticular purse string suture was applied by using a nonabsorbable and monofilament suture and a sliding arthroscopic knot was applied to both ends. Arthroscopic suture was shift 1 cm forward every day. In group 2 skin defect was leaved open and daily dressing was made and in both group defect diameters were measured every day and noted.

**Results:**

The skin defects were closed totally after 15 days in group 1 but in group 2 defects were reduced but still had a mean 1,5-cm diameter sircular defect.

**Conclusion:**

Closing large circular wound with purse string suture and gradual tightening decreases the healing time and expand the skin tissue without using any tissue expander.

## Introduction

Wound healing is an intricate process in which the skin (or another organ-tissue) repairs itself after injury [1]. There are four options for treating the wound: healing by second intention, primary closure, closure with local skin flap and closure with skin grafting [2, 3]. Primary closure after skin expansion with expander may be another option for wound closure [4]. Immediate reconstruction is favoured except an area which heals likely well by second intention [2]. There are many closure techniques available to cutaneous surgeons. One of them is the purse-string suture which is used to provide complete or partial closure of round skin defects. Greenbaum and Radonich first introduced the purse-string closure, which was used to repair a cutaneous surgical defect [4]. This running suture can either be placed horizontally in the dermis (intradermal or subcuticular purse-string suture) or be passed through the epidermis and dermis with each stitch (cuticular purse-string suture) [5, 6]. Viscoelastic property of skin provides skin closure in purse string suturing. Gibson and Kenedi first described these viscoelastic properties in 1967; the main properties being creep and stress relaxation [2]. Thus the skin has remarkable molding properties that can be used to the surgeon′s advantage [7, 8]. However large defects may not be completely closed. When the suture is cinched and tied, tissue at the wound periphery advances circumferentially, resulting in a decreased wound diameter. The resulting defect may either be covered with a skin graft or be allowed to heal by second intention [9]. In our animal study; we closed skin defect with a simple suture material by using subcuticular purse string suture technique by progressively cinching wound.

## Methods

Animal Ethical Committee approval was obtained from Marmara University Medical Faculty Ethical Institution. Twenty Sprague-Dawrey rats (weights between 350 -400 gr) divided into equal groups (n = 10 rats). General anestesia was made with using 35 mg/kg intraperitoneal Ketamin hydrochlorid, then intramuscular sodium pentobarbital (18 mg/kg) was applied for anesthesia. All wounds were then dressed with a single layer of sterile gauze, and the rat was wrapped with elastic tape. After recovery from anesthesia, the animals were caged individually and allowed free access to food and water.

**Figure 1:**
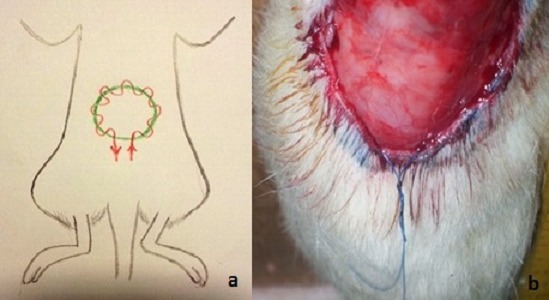
The skin defect and application of subcutaneous purse string is seen

**Figure 2:**
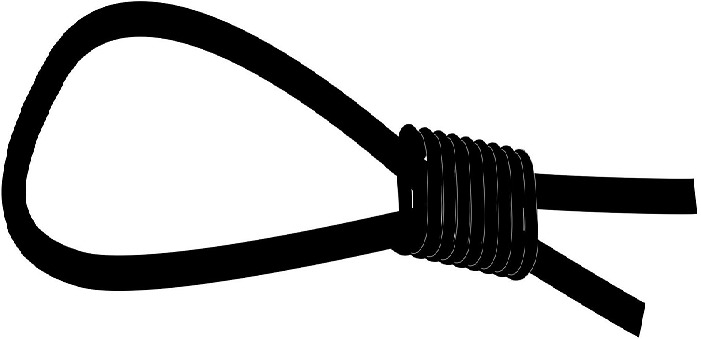
Drawing of Duncan loop knot

**Figure 3:**
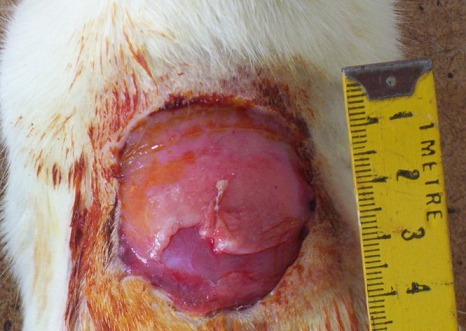
The skin defect in group 2 without suturing

**Surgical procedure**: The hairs at the dorsum of the rats were removed with using a electric razor. We created a circular skin defect, 4 cm in diameter on dorsum of the rats with using a plastic temple in both groups. In group 1, subcutaneous purse string suture was applied all around the edge of the wound by using a nonabsorbable and monofilament suture (PROLENE TM Polyprolene suture) (Figure 1). Two ends of the suture was tightened with a sliding knot, a Duncan loop (Figure 2). In group 2, the skin defect, suture and the knot was made in same way but the wound left for a secondary healing (Figure 3). In post operative period, intramuscular antibiotic prophylaxis was given to both of the groups for 5 days.

**Shrinking of the skin defects**: We applied daily dressing to wounds in both groups by irrigation with 0.9% saline and covering with sterile gauze; the rat was wrapped with elastic tape after the dressing. In group 1, during the change of daily dressing the wound was shrinked by sliding the knot 1 cm every day. In group 2, only daily dressing was made with same way in group 1. The diameter of the lesions were measured with using a caliper and a photo was taken daily for documentation.

**Statistical Analysis**: T test was used for comparison of open wound area between two groups. Repeated measures ANOVA were used for intragroup open wound area differences. Non-parametric analog test was used in case of exceeding the normal value. Ten animals per group were found sufficient for 80% power.

## Results

The skin defects were closed totally in 13 days in group 1 on the other hand the mean diameter of skin defect in group 2 was 1,5-cm (p < 0,001). There were no infection or suture related complication in group 1. No complication was observed in group 2 as well.

## Discussion

Purse-string suture technique has been used for the restoration of defects by many surgeons and is particularly suited to circular defects [2]. There are two types of purse-string suture technique: Intradermal purse-string closure and Cuticular purse-string closure. The intradermal (or subcuticular) purse string suture is performed by passing the running suture horizontally in the dermis before it is pulled taut [10]. Purse-string suture technique allows almost complete or partial closure of the defect by advancing the skin from the entire periphery of the wound [2, 11].

The purse-string suture closure have some advantages such as; it is a simple technique it doesn't need a special training, experience and it doesn't require a operation room also can be applied with several simple materials [2, 12]. It is inexpensive and the final results are often good, especially in lax skin area. Besides angiogenesis is promoted with tension [6, 13]. This procedure well suited for elderly patients, with loose skin [14, 15]. This tecnique also have some disadvantages such as; the suture has to be retained for a long time. Allergic contact dermatitis, alopecia, exuberant granulation tissue, hypertrophic scar, postoperative pain, wound dehiscence are other problems which are seen with this technique [6, 12, 16–18]. Certain areas of the body present unique and difficult challenges such as the shoulder and back. However less mobile areas of the body with high excess skin laxity are more sutibale for cosmetically healing with optimal scars. By using the purse-string suture in problematic less skin laxity areas, the overall length of the scar can be minimized [11].

However the large wounds may not be suitable for primary closure. Capability for primary closure depends on viscoelasticity of the periperal tissue of wound. Gibson and Kenedi first described these viscoelastic properties in 1967; the main properties being creep and stress relaxation [2, 19]. Mechanical creep occurs when a constant force stretches a piece of skin, when a piece of skin is stretched for a given distance and that distance is held constant; stress relaxation occurs and the force required to keep it stretched gradually decreases. This process occurs over minutes or hours [2, 20]. However during surgery there is no sufficient time for maximum expansion to close big defects. On the other hand a tight skin closure decreases blood circulation to the skin edges, thereby causing the tissues to become ischemic and cause skin death. Skin death results in a wound that is larger than the initial wound [21]. In our study; we closed skin defect with purse string suture technique by stretching the wound daily. So we can close larger defect than closeable at a time. So mechanical complication of excessive traction such as unacceptable cosmetic appearance, tissue necrosis and deforming the surrounding structures can be avoided.

A wound that is more than 6 hours old is also not suitable for primary repair. The risk of infection is greatly increased after this amount of time has lapsed. Wound with empty space also insuitable for primary closure. Empty space which occurs due to loss of subcutaneous tissue or swelling of the skin around the wound. At primary closing of wound with empty space, blood may collect at empty space which increasing the likelihood of infection and problems with wound healing [22]. In such cases, secondary healing is preferred. Another advantage of our technique is during shrinking it also permits a secondary wound healing. So while gradual wound closing dead space also is filled with granulation and dirty tissues are cleaned with repeated dressing. So our technique rapid the wound closure so decreases the cost and the healing time according to the secondary healing. Animal choice may be a negative point for our study because rat dorsal skin is so lax. Reviewer may say that tissue gaining is due to this laxity not due to creep -stress relaxation period. However we didn't have another choice at our animal lab.

Another technique for closing large defect is expansion and later closing with tissue expander. The primary objective of tissue expander is gaining extra skin tissue. However it brings some minor and major complication such as pain, seroma, dog ears, widening of scars as minor complications and infection, expander exposure, implant failure, induced ischemia as major complication [3, 23, 24]. Sometimes wounds are complicated due to this complication. In our technique, skin tissue peripheral to wound is expanded with time as a result of creep and stress relaxation so complication due to tissue expander will be avoided.

## Conclusion

As a result, closing large wound with purse string suture and gradual tightening decreases the healing time for wound which is not suitable for primary closing such as large wound, dirty wound or tight wound according to secondary healing. Moreover tissue is expanded without using any tissue expander.
